# Durability of Steel Bridge Deck Paving Materials Under Salt Attack in Coastal Hot–Humid Environments

**DOI:** 10.3390/polym18121519

**Published:** 2026-06-18

**Authors:** Yujie Zhang, Xiong Lan, Zhenqiang Han, Lei Zhu, Peidong Du, Zaiqin Chen, Aimin Sha

**Affiliations:** 1 School of Highway, Chang’an University, Xi’an 710064, China; 2024021099@chd.edu.cn (Y.Z.); jasonhan029@126.com (Z.H.);; 2 Guangxi Transportation Investment Group Co., Ltd., Wuzhou Expressway Operation Co., Ltd., Wuzhou 543300, China; 3 Guangxi Rongwu Expressway Co., Ltd., Wuzhou 543300, China; 4 Key Laboratory for Special Area Highway Engineering of Ministry of Education, Chang’an University, Xi’an 710064, China

**Keywords:** steel bridge deck paving materials, salt attack, hygrothermal salt-water cycling, indirect tensile strength, four-point bending fatigue, durability

## Abstract

Steel bridge deck pavements in coastal hot–humid regions are often exposed to the combined effects of moisture, salt, and temperature, which can accelerate material deterioration and shorten service life. To clarify the durability behavior of typical paving materials under such conditions, a comparative study was conducted on three asphalt mixtures used for steel bridge deck pavements: epoxy asphalt mixture (EA-10), dense-graded asphalt mixture (AC-13), and stone mastic asphalt mixture (SMA-10). The mixtures were subjected to hygrothermal salt-water cycling using a mixed chloride-sulfate solution, and their durability was evaluated through air void content, indirect tensile strength, and four-point bending fatigue tests. The results showed varying degrees of deterioration. The air void content of AC-13 increased by about 41.4% after 28 d at 60 °C, suggesting greater susceptibility to internal void damage under severe conditioning. The indirect tensile strength also decreased with wet–dry cycling; at 60 °C and 28 d, the strength retention of EA-10 remained 76.9%, higher than those of AC-13 and SMA-10. After conditioning at 60 °C, the fitted slope of fatigue life for SMA-10 reached −0.0052, compared with −0.0044 for AC-13 and 0.0027 for EA-10, indicating that SMA-10 was the most sensitive to hygrothermal salt attack, whereas EA-10 was the least affected. Overall, the resistance to hygrothermal salt-water damage followed the order EA-10 > AC-13 > SMA-10. The findings help clarify the durability behavior of steel bridge deck paving materials in coastal environments and provide support for durability-oriented material selection.

## 1. Introduction

Steel bridges have been widely used in long-span bridges and major transportation infrastructures because of their light self-weight, high spanning capacity, and rapid construction [1,2]. As the functional layer directly exposed to traffic loading and environmental actions, steel bridge deck pavements are closely related not only to driving safety and riding comfort, but also to the stress state of the steel deck plate and the long-term service performance of the whole bridge system [3,4]. Compared with conventional asphalt pavements, steel bridge deck pavements are characterized by a thinner paving layer, a stiffer substrate, and stronger interfacial restraint. Under repeated traffic loading, they are therefore more prone to distresses such as cracking, debonding, and raveling [5,6]. As a result, paving materials used on steel bridge decks are expected to provide not only good rutting resistance, crack resistance, and fatigue resistance, but also sufficient environmental durability for long-term service [7,8].

The service environment in coastal hot–humid regions further increases the durability challenge for steel bridge deck paving materials. Compared with inland areas, these regions are exposed for long periods to the combined effects of high temperature, high humidity, and salt attack. Salts carried by sea fog, rainfall, and humid air can enter pavement materials together with moisture and accumulate during repeated wetting and drying, thereby accelerating performance deterioration. Zhang and Huang reported that the high salt-fog concentration and hot–humid climate in coastal areas can significantly accelerate the aging of asphalt materials and promote the development of internal damage through salt crystallization [9]. Jiang et al. also pointed out that salt, moisture, and temperature in coastal environments do not act independently, but rather interact in a coupled manner, making durability problems in asphalt pavements more complicated [10,11,12]. At the mixture level, Zhang et al. found that sea salt solution can gradually penetrate into asphalt mixtures and reduce their strength and moisture stability, while Li et al. showed that the mechanical properties of asphalt mixtures continuously decline under large hygrothermal cycling conditions [13]. In addition, Wang et al., in a recent review, summarized that salt-induced deterioration in asphalt materials is mainly reflected in pore structure evolution, interfacial weakening, and deterioration of the asphalt mastic, all of which eventually lead to a reduction in strength and durability [14]. These findings suggest that the durability problem of steel bridge deck paving materials in coastal hot–humid regions is essentially a coupled deterioration process involving salt, moisture, and temperature, and therefore deserves targeted investigation [15,16].

In recent years, research on steel bridge deck paving materials has expanded from simple material selection to broader issues involving structural forms, pavement performance, and fatigue cracking behavior. Chen et al. pointed out in a review that research on steel bridge deck pavements has covered aggregate gradation design, high-temperature stability, low-temperature cracking resistance, moisture stability, and fatigue fracture behavior, and that the corresponding test methods have gradually developed from conventional pavement performance tests to more targeted approaches such as indirect tensile testing, overlay testing, and fatigue evaluation of composite systems [17]. For commonly used bridge deck paving materials, Lu and Bors reviewed the application of epoxy asphalt in orthotropic steel bridge deck pavements and concluded that it shows clear advantages in rutting resistance, fatigue resistance, and resistance to moisture- and salt-related damage, which has supported its use in a number of long-span steel bridges [18]. Ren et al. compared the fracture resistance of EA, AC, and SMA mixtures under severe environmental conditions through three-point bending and semicircular bending tests, and showed that environmental exposure can continuously reduce the cracking resistance of asphalt mixtures, with clear differences among materials [19,20]. Besides fracture behavior, fatigue has also been recognized as one of the key issues governing the durability of bridge deck pavements. Meng et al. emphasized that the four-point bending fatigue test has become an important method for evaluating the fatigue performance of asphalt mixtures because it can better simulate the bending tensile stress state in service [21]. Abhijith and Narayan further noted that the modulus obtained from four-point bending testing is essentially an apparent response resulting from damage development at different locations within the beam, highlighting the structural nature of fatigue damage evolution [22]. Among the mixtures commonly used in steel bridge deck pavements, epoxy asphalt mixture, stone mastic asphalt mixture, and dense-graded asphalt mixture are typical choices. These mixtures differ in binder system, aggregate gradation, and internal structure, and therefore may respond differently to environmental exposure. In coastal hot–humid service environments, steel bridge deck paving materials are subjected to the combined influence of salt attack, moisture, temperature, and repeated traffic loading. Under these conditions, void development, interfacial weakening, strength loss, and fatigue damage do not occur independently, but tend to evolve together during service. For this reason, hygrothermal salt-water cycling was considered in this study as a representative environmental condition for examining the durability behavior of typical steel bridge deck paving mixtures in coastal regions. Although previous studies have discussed moisture damage, salt erosion, cracking resistance, or fatigue behavior, these issues have mostly been treated separately. As a result, the durability response of steel bridge deck paving materials under coastal hygrothermal salt exposure is still not fully understood. In particular, there is still a lack of a more direct link among volumetric change, tensile strength degradation, and fatigue deterioration under a consistent experimental framework. This makes it difficult to clearly identify how different paving mixtures respond to the same coastal service-related condition.

In view of this, three asphalt mixtures commonly used in steel bridge deck pavements, namely epoxy asphalt mixture (EA-10), dense-graded asphalt mixture (AC-13), and stone mastic asphalt mixture (SMA-10), were selected in this study. A mixed chloride-sulfate salt solution was used to simulate salt exposure in coastal regions, and a hygrothermal cycling procedure was adopted to represent the combined effects of salt, moisture, and temperature during service. On this basis, the durability behavior of the three mixtures was examined from three aspects, namely volumetric evolution, tensile strength degradation, and fatigue deterioration, through air void measurement, indirect tensile testing, and four-point bending fatigue testing. This study aims to clarify the deterioration characteristics of typical steel bridge deck paving materials under coastal hot–humid salt exposure. The results can provide a practical reference for durability-oriented material selection and performance assessment of steel bridge deck pavements in coastal regions.

## 2. Materials and Methods

### 2.1. Materials

Three asphalt mixture types commonly used in steel bridge deck surfacing were selected in this study, namely epoxy asphalt mixture, stone mastic asphalt mixture, and dense-graded asphalt mixture. To ensure the comparability of test results, the three mixtures were prepared using the same aggregate system from the same source. Basalt was used as coarse and fine aggregate, and limestone powder was used as mineral filler. The physical properties of the aggregates and filler satisfied the relevant specification requirements, and the key indices are listed in Table 1. For binders, the epoxy asphalt mixture used an epoxy asphalt binder system consisting of Shell 70# paving asphalt, epoxy resin component A, and curing agent component B, with a mass ratio of 100:56:44 [23]. The AC and SMA mixtures used styrene–butadiene–styrene (SBS)-modified asphalt as the binder. The main physical properties of the binders were determined by conventional tests and are summarized in Table 2. In addition, lignin fiber was incorporated into the SMA mixture as a stabilizing additive to maintain mixture stability during mixing and compaction. The basic properties of the lignin fiber are provided in Table 3.

### 2.2. Mixture Design

**Figure 1:**
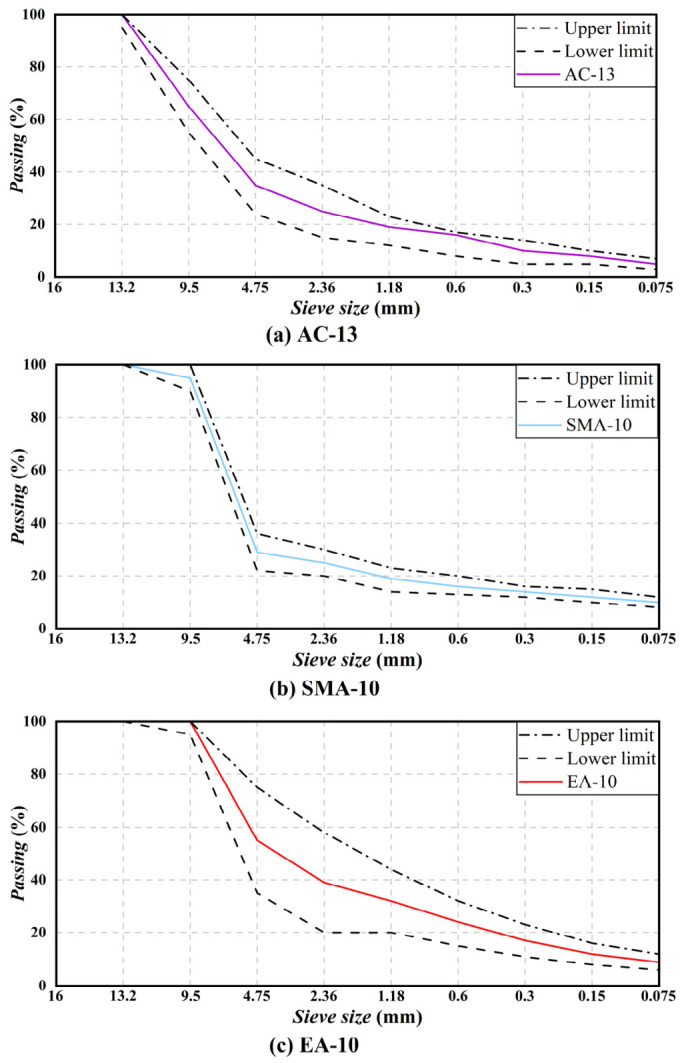
Aggregate gradations of three asphalt mixtures: (**a**) AC-13; (**b**) SMA-10; (**c**) EA-10.

In this study, the gradation types of the three asphalt mixtures were selected as EA-10, SMA-10, and AC-13. The aggregate gradation for each mixture was designed in accordance with the relevant specifications, and the corresponding gradation curves are presented in Figure 1. Similar practice of reporting mixture proportions and gradation curves has been widely adopted in the literature. The optimum asphalt content, *OAC*, of each mixture was determined through laboratory mix-design procedures and was further checked using volumetric parameters, and the final *OAC* values together with the key volumetric indices are summarized in Table 4. For SMA-10, lignocellulosic fiber was incorporated as a stabilizer at 0.3 percent by total mixture mass to enhance mixture stability during mixing and compaction and to mitigate binder draindown [24]. After compaction, EA specimens were subjected to a curing procedure prior to salt-solution conditioning and mechanical testing to ensure that the epoxy asphalt system fully reacted and reached a stable mechanical state. The use of epoxy materials is closely associated with the formation of a cured three-dimensional network structure, which provides a mechanistic basis for applying a dedicated curing protocol for epoxy-based systems [25].

### 2.3. Salt-Solution Preparation

To simulate the coastal hot–humid regions, a mixed chloride-sulfate salt solution was used as the erosion medium. The solution was prepared by dissolving sodium chloride and sodium sulfate in water at a mass ratio of 8 to 1, and the basic technical indicators of the two salts are listed in Table 5. This choice was intended to reflect the coexistence of chloride and sulfate ions in marine and coastal salt exposure scenarios reported in previous studies. In addition, seawater salinity is often taken as about 3.5 percent by mass [26], while higher salinities such as 5 percent and 13 percent are commonly adopted in laboratory work to intensify damage and improve discrimination among materials. Therefore, the total salt mass fraction was set to 10 percent in this study, and the prepared solution was used for subsequent salt-immersion erosion tests [27].

## 3. Experimental Programs

### 3.1. Hygrothermal Cycling Tests

In the present work, specimens were conditioned in the prepared salt solution under different temperature conditions to simulate the coupled effects of thermal exposure and cyclic wetting–drying in coastal bridge deck environments. The temperatures were set at 20 °C, 40 °C, and 60 °C to represent normal-, medium-to-high-, and high-temperature conditions commonly experienced by steel bridge deck pavements during daily service. Each cycle consisted of two stages: the specimens were first immersed in the salt solution for 12 h to represent the wetting period, and they were then removed and oven-dried for 12 h to represent the surface-drying period. This 12 h immersion plus 12 h drying protocol has also been used in salt-related wet–dry cycling studies to ensure fully wet and fully dry states within one cycle. The conditioning protocol was designed as an accelerated laboratory salt-corrosion procedure for comparing the durability of different mixtures. A total of 28 cycles were applied to capture the progressive deterioration process within a practical test period, and specimens were tested at 7-cycle intervals to track the evolution of mixture performance [28].

### 3.2. Vv Test

**Figure 2:**
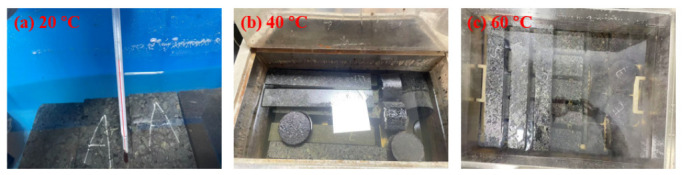
Test specimens under different wet heat cycle conditions: (**a**) 20 °C; (**b**) 40 °C; (**c**) 60 °C.

The air void content (*VV*) of asphalt mixtures was determined using the drainage method based on the bulk specific gravity and the theoretical maximum specific gravity. The *VV* measurements were conducted for specimens after completing the hygrothermal cycling at three temperature levels (20 °C, 40 °C, and 60 °C). Representative photographs of the conditioning at these temperatures are presented in Figure 2a–c. Specifically, the bulk specific gravity of compacted specimens was measured by the saturated surface-drying and submerged weighing procedure following ASTM D2726, while the theoretical maximum specific gravity was obtained in accordance with ASTM D2041. *VV* was then calculated using the bulk and maximum specific gravity values according to Equations (1) and (2). For each conditioning case, three replicate specimens were prepared and tested, and the average value was reported as the final result. After the *VV* determination, the same specimens were subsequently used for the indirect tensile (IDT) test.

(1) $$G_{mb}=\frac{m_{d}}{m_{ssd}-m_{w}}$$

(2) $$VV=\left(1-\frac{G_{mb}}{G_{mm}}\right)\times 100\%$$

where *m_d_* is the mass of the specimen in air (g); *m_ssd_* is the saturated surface-dry mass of the specimen (g); *m_w_* is the mass of the specimen in water (g); *G_mb_* is the bulk specific gravity of the mixture; and *G_mm_* is the theoretical maximum specific gravity of the mixture.

### 3.3. Indirect Tensile Test

**Figure 3:**
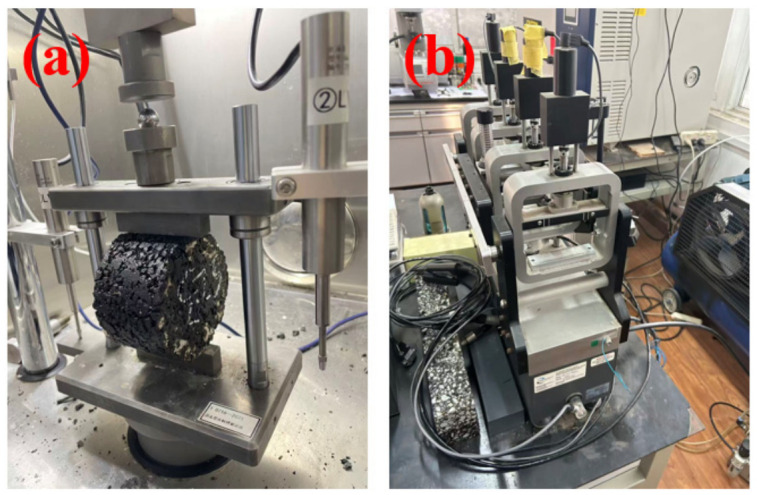
Test apparatus: (**a**) IDT test; (**b**) four-point bending fatigue test.

After the VV measurement described in Section 3.2, the same specimens were subsequently subjected to the indirect tensile (IDT) test to determine their indirect tensile strength (ITS) after salt-induced hygrothermal cycling. Cylindrical Marshall specimens were cut into disk specimens with a diameter of 101.6 mm and a thickness of 63.5 mm. The IDT test was conducted in accordance with ASTM D6931 using an HTHY-STM50A multifunctional asphalt mixture testing system (Beijing Hangtian Hongyu Testing Equipment Technology Co., Ltd., Beijing, China). The loading configuration is shown in Figure 3a. Prior to loading, the specimens were immersed in a 10% salt solution at 15 °C for no less than 1.5 h to ensure full wetting, and then equilibrated at 15 °C until a stable internal temperature was reached [29]. The test was performed at 15 °C under displacement control with a loading rate of 50 mm/min until failure, and the peak load was recorded. A Poisson’s ratio of 0.30 was adopted as recommended in ASTM D6931. The IDT strength was calculated using Equation (3). For each conditioning case, three replicate specimens were tested, and the average value was reported.

(3) $$ITS=\frac{2P}{\pi Dt}$$

where *P* is the peak load at failure (N); *D* is the specimen diameter (mm); and *t* is the specimen thickness (mm).

### 3.4. Four-Point Bending Fatigue Test

The four-point bending fatigue test was conducted to evaluate the fatigue resistance of asphalt mixtures after salt-induced hygrothermal conditioning. Because the 60 °C condition represented the most severe hygrothermal exposure in this study, the fatigue evaluation was carried out for specimens conditioned at this temperature. The test was carried out using an four-point bending beam (FPBB) fatigue testing system manufactured by IPC Global Pty Ltd., Melbourne, Australia, and a representative photograph of the apparatus is shown in Figure 3b. The specimens were tested under strain-controlled loading to better reflect the strain-driven response of asphalt layers subjected to repeated traffic actions. A target tensile strain level of 400 με was adopted, which was selected to represent the bending strain demand induced by medium-duty vehicle traffic on coastal roadways [30]. The test temperature was maintained at 20 °C to provide a stable reference condition for comparing fatigue degradation after conditioning. During the test, the stiffness evolution of the beam specimen was continuously monitored as a function of loading cycles. Following ASTM D7460-10, the reference stiffness *S*_0_ was taken as the stiffness measured at the 50th cycle, and the fatigue life *N_f_* was defined as the number of cycles corresponding to a 50% reduction in stiffness relative to *S*_0_.

## 4. Results and Discussion

### 4.1. Evolution of Vv Under Hygrothermal Salt Cycles

**Figure 4:**
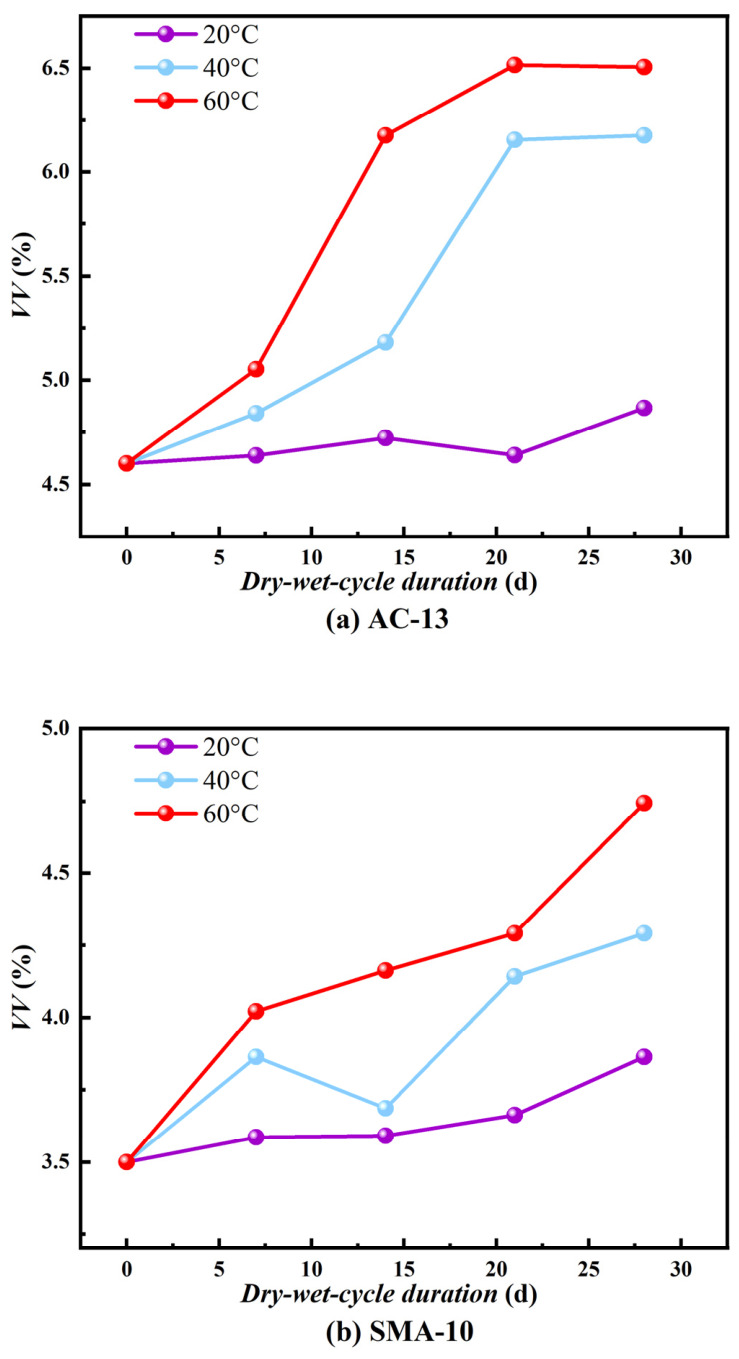

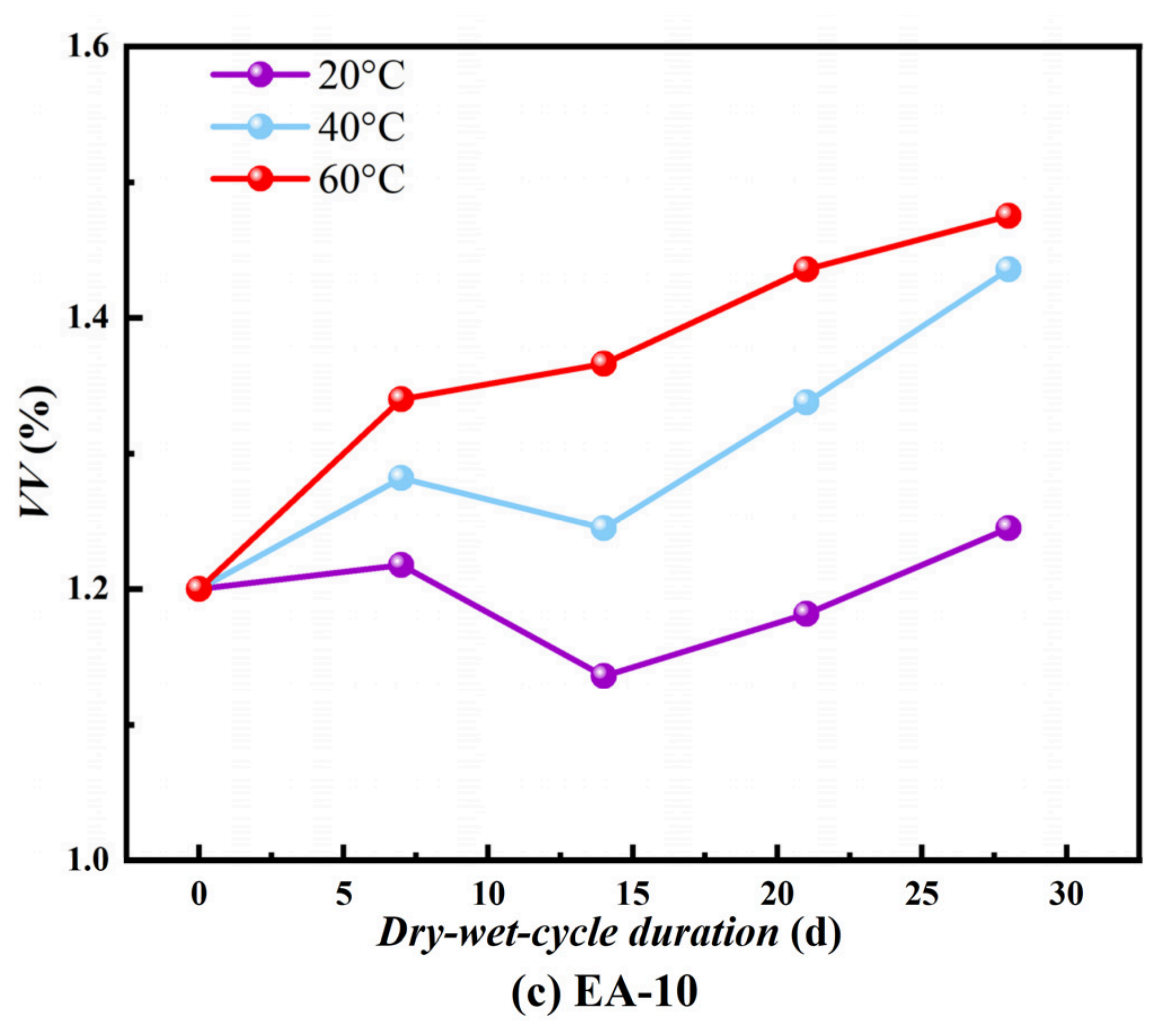
Changes in VV of asphalt mixture under hygrothermal salt-water cycling: (**a**) AC-13; (**b**) SMA-10; (**c**) EA-10.

Figure 4a–c show the changes in air void content (*VV*) of AC-13, SMA-10, and EA-10 under hygrothermal salt cycling at 20 °C, 40 °C, and 60 °C. A 10% salt solution was used as an accelerated laboratory salt-corrosion condition. It reflects the coexistence of chloride and sulfate ions in coastal regions, while the relatively high salt concentration helps distinguish the durability responses of different mixtures within a practical test period. For all three mixtures, *VV* generally increases with cycling duration, and the increase becomes more obvious at higher temperature. This suggests that the hot–humid saline environment may promote moisture and salt ingress through pores and weak interfacial regions, thereby causing progressive damage to the internal void structure [31]. A clearer comparison can be made by looking at the *VV* increase at 28 d relative to 0 d. For AC-13, the change at 20 °C is limited, with an increase of only about 5.8%, whereas the increase becomes much larger at 40 °C and 60 °C, reaching about 34.3% and 41.4%, respectively. This indicates that the void structure of AC-13 is particularly sensitive to elevated temperature under hygrothermal salt exposure. SMA-10 shows a similar tendency, although the overall change is less abrupt, with *VV* increases of about 10.4%, 22.6%, and 35.5% at 20 °C, 40 °C, and 60 °C, respectively. EA-10 keeps the lowest absolute *VV* level, around 1.2%, throughout the test. Based on the corrected values, its *VV* increases by about 3.8% at 20 °C, and by about 19.7% and 22.9% at 40 °C and 60 °C. Although EA-10 still undergoes measurable void growth under hygrothermal salt cycling, the increase remains comparatively limited, which points to better structural stability [20,32].

This difference is consistent with the characteristics of the three mixtures. After curing, EA-10 forms a relatively dense three-dimensional crosslinked network, which improves the integrity of the binder system and makes the rapid development of interconnected voids less likely. Under the same conditioning, its *VV* therefore changes less. By comparison, SBS-modified mixtures are more likely to experience progressive interfacial weakening and local micro-damage under repeated moisture–salt action, and this becomes more evident at elevated temperature. It should also be noted that EA-10 still shows nearly 20% relative *VV* growth at 40–60 °C, which means that prolonged exposure to a hot hygrothermal salt environment can still alter its internal structure, even though the extent of change is smaller than that of the other two mixtures [33,34]. The time-dependent pattern is also different among the mixtures. At 60 °C, AC-13 develops most of its total *VV* increase within 0–14 d, accounting for about 83% of the overall increment, suggesting that its internal void structure opens up mainly in the early stage. SMA-10 changes more gradually, with about 53% of the total increase occurring within 0–14 d. EA-10 falls between the two, with about 60% of the total increase occurring in the same period, indicating that it also responds noticeably to the early stage of high-temperature conditioning. The slight decrease observed for EA-10 during 0–14 d at 20 °C is very small and may be related to temporary pore filling caused by salt precipitation and/or normal experimental scatter, without affecting the overall increasing trend with cycling duration [35]. Taken together, the larger VV growth at higher temperature suggests that moisture–salt transport paths are more easily formed and maintained, which may in turn contribute to the subsequent reductions in IDT strength and fatigue performance.

### 4.2. Degradation of ITS Under Hygrothermal Salt Cycles

**Figure 5:**
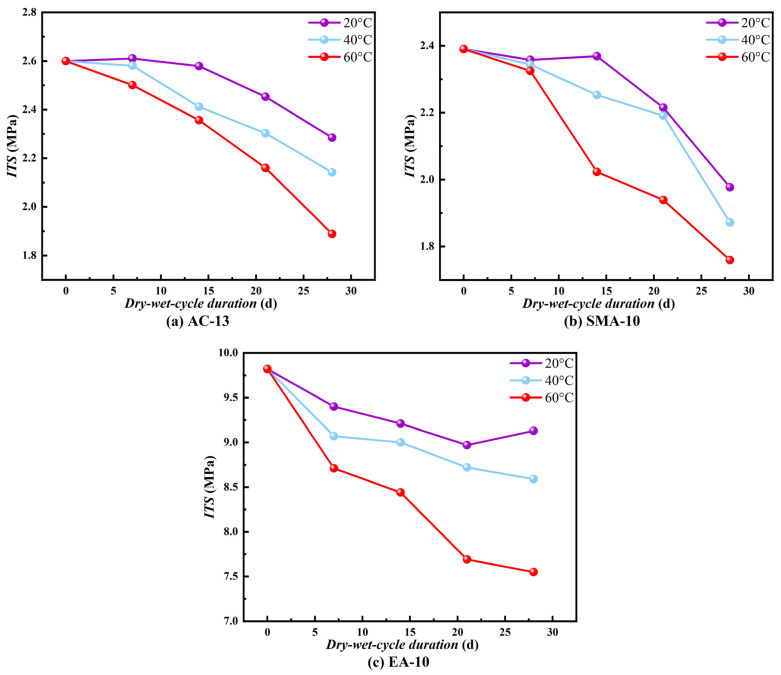
Changes in ITS of asphalt mixture under hygrothermal salt-water cycling: (**a**) AC-13; (**b**) SMA-10; (**c**) EA-10.

Figure 5 presents the changes in ITS splitting strength of AC-13, SMA-10, and EA-10 under hygrothermal salt cycling at 20 °C, 40 °C, and 60 °C. For all three mixtures, the splitting strength decreases with increasing cycling duration, and the reduction becomes more evident at higher temperature. This indicates that the hot–humid saline environment continuously weakens the tensile resistance of asphalt mixtures. In terms of absolute strength level, EA-10 remains much stronger than the two SBS-modified mixtures throughout the test. Its initial strength is 9.82 MPa, and even after 28 d at 60 °C it still reaches 7.55 MPa, which is about 4.0 times that of AC-13 and 4.3 times that of SMA-10 under the same condition. This clear advantage is related to the epoxy asphalt system, where curing forms a relatively dense three-dimensional crosslinked network and gives the material higher cohesion and structural integrity, allowing it to retain a comparatively high tensile resistance during hygrothermal salt exposure [36]. At the same time, the reduction in EA-10 is still noticeable at high temperature. At 60 °C, for example, its strength decreases by 2.27 MPa from 0 to 28 d, showing that a high initial strength does not mean that the material is unaffected by severe hygrothermal salt attack [37]. To compare durability more directly, the strength retention can also be considered. At 28 d, the splitting strengths of AC-13 are 2.285, 2.142, and 1.889 MPa at 20 °C, 40 °C, and 60 °C, corresponding to retentions of 87.9%, 82.4%, and 72.7%, respectively. The corresponding values for SMA-10 are 1.977, 1.872, and 1.759 MPa, with retentions of 82.7%, 78.3%, and 73.6%. For EA-10, the strengths are 9.13, 8.59, and 7.55 MPa, corresponding to retentions of 93.0%, 87.5%, and 76.9%. These results show that EA-10 retains its strength better at 20–40 °C. Once the temperature rises to 60 °C, however, the retention drops markedly for all three mixtures, and the differences among them become smaller, indicating that elevated temperature strongly amplifies salt-induced damage.

The temperature effect can also be seen from the strength gap between temperatures. At 28 d, the strength at 60 °C is lower than that at 20 °C by 0.396 MPa for AC-13, 0.218 MPa for SMA-10, and 1.58 MPa for EA-10. Expressed in relative terms, the strength ratios of 60 to 20 °C at 28 d are about 82.7%, 89.0%, and 82.7%, respectively. This suggests that SMA-10 has a smaller end-stage temperature gap, whereas the additional loss in EA-10 becomes more evident at high temperature. For EA-10, the larger absolute gap is also associated with its much higher initial strength level, which makes the temperature-related loss more visible in absolute terms [38]. The timing of deterioration is also different among the mixtures, especially at 60 °C. EA-10 shows a more obvious early-stage drop: its strength decreases from 9.82 MPa to 8.44 MPa during 0–14 d, a loss of 1.38 MPa, and then decreases by a further 0.89 MPa during 14–28 d. AC-13 shows a different pattern, with a smaller loss of 0.244 MPa during 0–14 d but a larger loss of 0.467 MPa during 14–28 d, indicating that deterioration becomes more apparent at later ages. SMA-10 lies between these two cases, with losses of 0.367 MPa during 0–14 d and 0.264 MPa during 14–28 d, giving a more even decline over time. This difference is in line with the void evolution discussed in Section 4.1, where mixtures showing more persistent development of voids and transport paths are also more likely to experience continued strength loss at later stages [34]. It should also be noted that the EA-10 data at 20 °C do not decrease strictly monotonically. The strength is 8.97 MPa at 21 d and then increases slightly to 9.13 MPa at 28 d, giving a rebound of 0.16 MPa. Since the magnitude is small, this is more likely due to normal experimental scatter, although it may also be related to stress relaxation or local structural stabilization in the cured epoxy system under prolonged isothermal wet exposure. In any case, this small fluctuation does not change the overall trend, namely that EA-10 decreases continuously at 40 °C and 60 °C, with the most severe deterioration occurring at 60 °C [32,39]. Overall, the IDT results show that hygrothermal salt cycling reduces the tensile resistance of all three mixtures, and that temperature plays a strong role in accelerating this process. The three mixtures differ not only in absolute strength level, but also in their retention, temperature sensitivity, and deterioration pattern with cycling duration [40].

### 4.3. Four-Point Bending Fatigue Curves of Asphalt Mixtures After Hygrothermal Salt-Water Cycling

**Figure 6:**
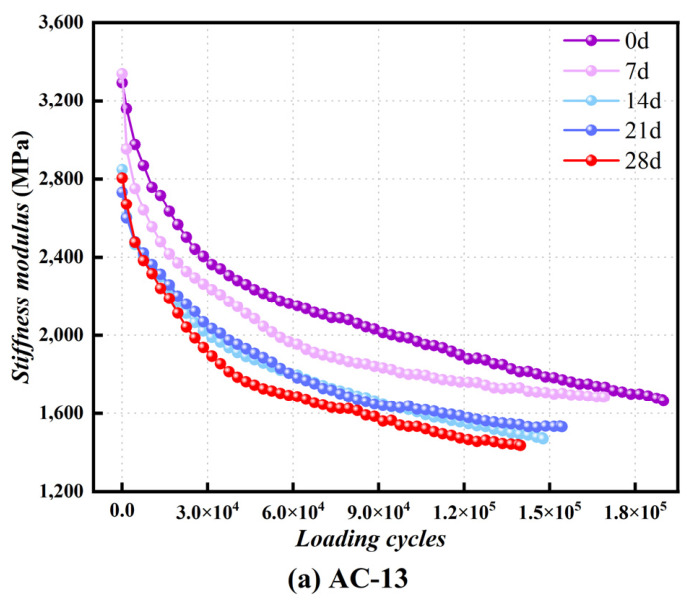

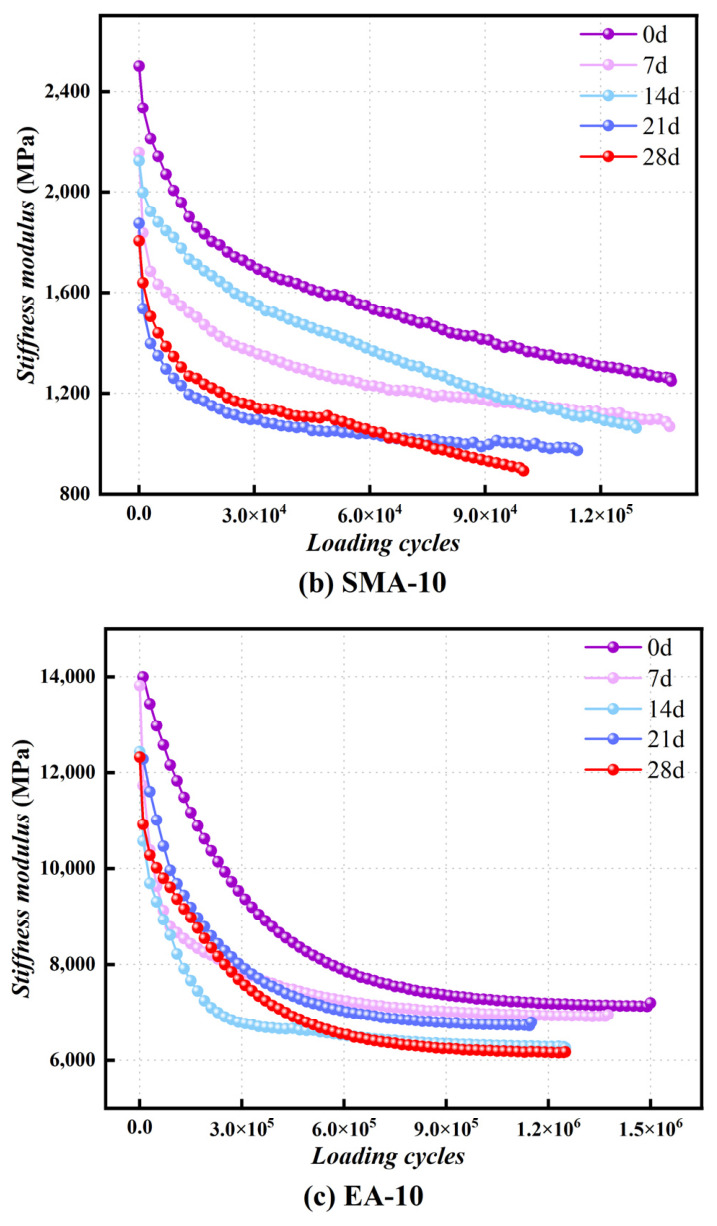
Four-point bending fatigue curves of asphalt mixtures after hygrothermal salt-water cycling: (**a**) AC-13; (**b**) SMA-10; (**c**) EA-10.

**Figure 7:**
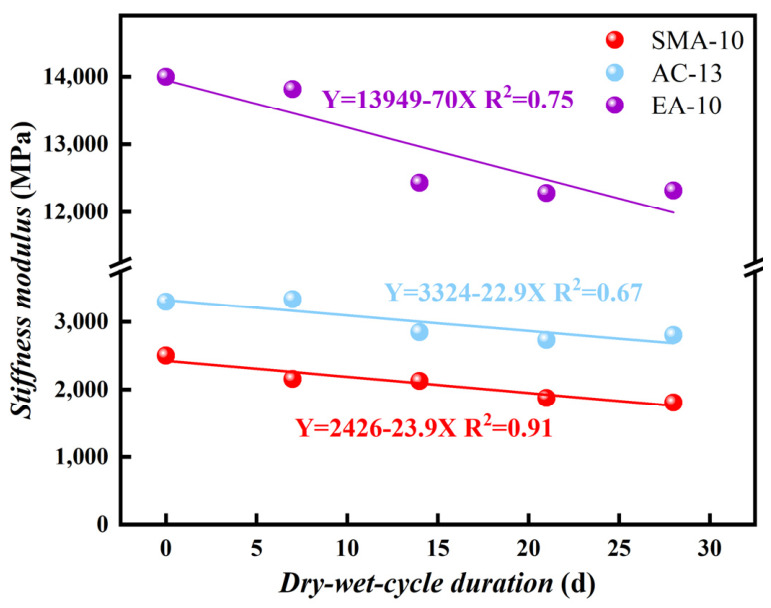
Changes in initial stiffness modulus of asphalt mixtures after hygrothermal salt-water cycling.

**Figure 8:**
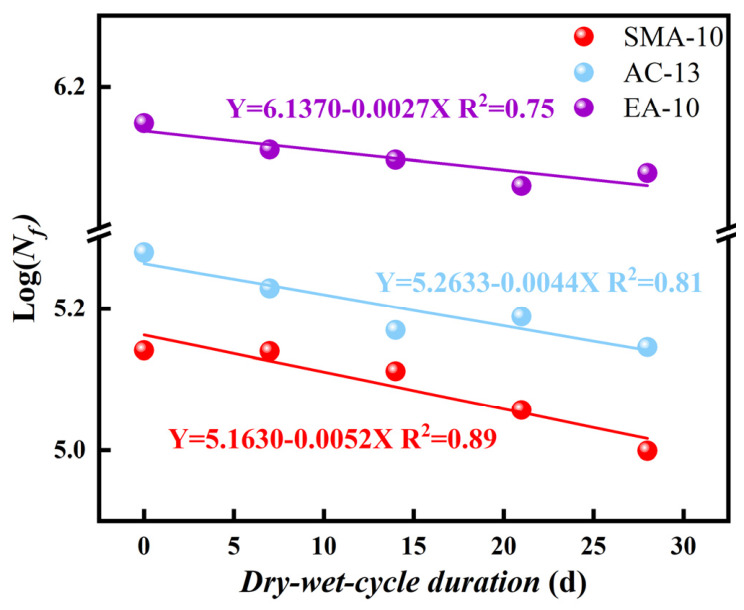
Changes in *N_f_* of asphalt mixtures after hygrothermal salt-water cycling.

Figure 6a–c present the stiffness modulus decay curves of AC-13, SMA-10, and EA-10 after hygrothermal salt-water cycling at 60 °C. Figure 7 and Figure 8 show the changes in the initial stiffness modulus *S*_0_ and fatigue life *N_f_* of the three mixtures after different wet–dry cycle durations at 60 °C. It can be seen that the four-point bending fatigue performance of all three mixtures deteriorated to different extents after hygrothermal salt-water cycling. This was mainly reflected in a reduction in the initial stiffness modulus, a shortening of fatigue life, and an overall earlier onset of stiffness decay. This agrees with the trend observed in the IDT results, namely that a high-temperature hygrothermal salt environment continuously weakens the mechanical performance of asphalt mixtures, although the degree of sensitivity varies among materials.

As shown in Figure 6, the stiffness decay curves of the three mixtures all exhibit a clear staged pattern. At the beginning of loading, the stiffness modulus drops rapidly, after which the decay rate gradually decreases and the material enters a relatively stable stage of damage accumulation. This suggests that, in the early stage of fatigue loading, pre-existing defects and the microcracks formed during environmental conditioning are the first to adjust and propagate, leading to an obvious loss of stiffness. As loading continues, damage develops in a more stable manner, and the slope of the curve decreases accordingly [41]. In terms of the overall trend, EA-10 shows a slower decay process at all conditioning ages, indicating that it is able to maintain better structural stability under bending fatigue loading. The difference between AC-13 and SMA-10 deserves more attention. Although SMA-10 has a stronger aggregate skeleton, its stiffness decay curve in Figure 6b is not superior overall. In particular, the curve falls faster at later wet–dry cycle ages, indicating that under the present test conditions, the skeleton advantage of SMA-10 did not translate into better fatigue performance. Figure 7 further shows the variation in the initial stiffness modulus *S*_0_ of the different mixtures. As the wet–dry cycle duration increased, *S*_0_ decreased for all three mixtures, but the magnitude of reduction was different. By 28 d, the decreases in *S*_0_, relative to the initial condition, were about 27.8%, 15.0%, and 12.0% for SMA-10, AC-13, and EA-10, respectively. In relative terms, SMA-10 exhibited the most pronounced loss of initial stiffness, followed by AC-13, while EA-10 showed the smallest reduction. The linear fitting results in Figure 7 support the same conclusion. The fitted slopes of SMA-10 and AC-13 were close, at −23.9 MPa/d and −22.9 MPa/d, respectively. Although EA-10 had a larger absolute slope of −70 MPa/d, this was mainly because its initial modulus was much higher than those of the other two mixtures. When judged on a relative basis, EA-10 remained the most stable of the three. Compared with the initial stiffness modulus, fatigue life responded more directly to environmental conditioning. Figure 8 shows that the fatigue life of all three mixtures decreased with increasing wet–dry cycle duration, but the extent of reduction was clearly different. EA-10 maintained the highest fatigue life throughout the test period, with a decrease of about 14.9% from 0 d to 28 d. The corresponding reductions were about 26.4% for AC-13 and 27.8% for SMA-10. In other words, under 60 °C hygrothermal salt-water cycling, EA-10 showed the best fatigue life retention, whereas SMA-10 showed the largest decrease in fatigue life. The fitted slopes were −0.0052, −0.0044, and −0.0027 for SMA-10, AC-13, and EA-10, respectively, again indicating that hygrothermal salt attack had a stronger effect on fatigue damage accumulation in SMA-10, while the effect on EA-10 was relatively smaller.

Another noteworthy result is that the fatigue life of AC-13 was consistently higher than that of SMA-10. This does not fully agree with the common impression that SMA performs better at high temperatures. However, when the present loading mode and conditioning environment are considered together, the result is understandable. The advantage of SMA-10 mainly lies in its stronger aggregate skeleton and better resistance to permanent deformation. In the four-point bending fatigue test, however, strain control was adopted. Under this mode, fatigue performance is not governed only by skeleton strength, but is also closely related to the energy dissipation capacity of the mastic system, the condition of the interfaces, and the ability of the internal structure to accommodate deformation. After 60 °C hygrothermal salt-water cycling, the asphalt film and aggregate–binder interface in SMA-10 were more severely weakened. Once local stress concentration developed, cracks could propagate more easily along the interface, which accelerated stiffness loss and fatigue life reduction. By contrast, AC-13 is a continuously graded mixture with a more uniform internal structure and a more continuous stress-transfer path during bending fatigue. As a result, its deformation compatibility after conditioning was relatively better. This is likely one of the main reasons why AC-13 showed a longer fatigue life than SMA-10 in the present study. It should be noted that this comparison applies to the present test conditions, namely 60 °C hygrothermal salt-water conditioning and strain-controlled loading at 400 με. EA-10, by comparison, remained relatively stable. In terms of both initial stiffness modulus and fatigue life, EA-10 was clearly superior to the other two mixtures. This is associated with its denser internal structure and the crosslinked network formed by epoxy asphalt [42]. The VV and IDT results presented earlier already showed that EA-10 experienced a smaller change in void structure under the hygrothermal salt environment and retained its strength more effectively. In the four-point bending fatigue stage, this structural advantage became more evident. Water and salt were less likely to form rapidly connected transport paths within the material, and both interface degradation and microcrack propagation were more effectively restrained [5]. As a result, stiffness decayed more slowly and fatigue life remained longer.

Overall, 60 °C hygrothermal salt-water cycling had an adverse effect on the fatigue performance of all three mixtures, although the extent of deterioration was clearly different. In terms of absolute fatigue performance, EA-10 performed best, AC-13 ranked second, and SMA-10 showed the poorest performance. In terms of environmental sensitivity, SMA-10 was affected the most, AC-13 was intermediate, and EA-10 was the least affected. Combined with the earlier air-void and IDT results, these findings suggest that under a hygrothermal salt environment, the evolution of internal void structure, the degradation of interfacial adhesion, and crack propagation under repeated fatigue loading are strongly coupled. Owing to its more stable internal structure, EA-10 showed a stronger ability to resist this coupled deterioration. The VV, IDT, and fatigue results show a consistent deterioration pattern. After 28 d of cycling at 60 °C, the VV increases of AC-13, SMA-10, and EA-10 were 41.4%, 35.5%, and 22.9%, respectively. The corresponding ITS losses were 27.3%, 26.4%, and 23.1%, while the fatigue life reductions were 26.4%, 27.8%, and 14.9%. These results suggest that the development of void damage is accompanied by a gradual loss of tensile resistance and fatigue performance. The three indicators therefore reflect different aspects of the same deterioration process under hygrothermal salt-water cycling.

## 5. Conclusions

To investigate the durability of steel bridge deck paving materials under salt attack in coastal hot–humid environments, AC-13, SMA-10, and EA-10 were examined through air void measurements, indirect tensile testing, and four-point bending fatigue testing. Based on the experimental results obtained under hygrothermal salt-water cycling, the following conclusions can be drawn:

1. Hygrothermal salt-water cycling increased the air void content of all three mixtures, and the increase became more pronounced with increasing temperature. AC-13 showed the largest void growth, SMA-10 exhibited an intermediate response, and EA-10 maintained the lowest void level and the smallest structural change.
2. The indirect tensile strength of all mixtures decreased with wet–dry cycling, and the reduction became more severe at higher temperature. Among the three mixtures, EA-10 maintained the highest strength and the best strength retention, while AC-13 and SMA-10 were more vulnerable to hygrothermal salt exposure.
3. The four-point bending fatigue performance of all mixtures deteriorated after hygrothermal salt-water cycling at 60 °C. Both the initial stiffness modulus and fatigue life decreased with increasing cycle duration. EA-10 maintained the highest stiffness level and the longest fatigue life, whereas SMA-10 showed the strongest sensitivity to conditioning.
4. Under the present test conditions, the overall resistance to hygrothermal salt-water damage followed the order EA-10 > AC-13 > SMA-10. After 28 d of cycling at 60 °C, the VV increases of AC-13, SMA-10, and EA-10 were 41.4%, 35.5%, and 22.9%, respectively, accompanied by strength losses of 27.3%, 26.4%, and 23.1% and fatigue life reductions of 26.4%, 27.8%, and 14.9%. These results show that void growth is closely related to mechanical deterioration under hygrothermal salt exposure.

Taken together, the results show that salt attack in coastal hot–humid environments can substantially accelerate the deterioration of steel bridge deck paving materials, although the extent of deterioration depends strongly on mixture type. These results provide a useful basis for the selection and durability assessment of steel bridge deck paving materials in coastal hot–humid regions. Future work should consider a wider range of environmental conditions and incorporate microstructural characterization to further clarify the relationships among void evolution, interfacial damage, and fatigue deterioration.

## Figures and Tables

**Table 1:** Properties of basalt aggregate and limestone powder.

Materials	Properties	Unit	Results	Specifications
Aggregate	Bulk specific gravity	g·cm^−3^	2.926	ASTM C127
	Aggregate crushing resistance	%	9.1	ASTM C131
	Los Angeles abrasion value	%	11.2	ASTM C535
	Flat and elongated particles	%	3.8	ASTM D4791
	Water absorption	%	0.61	ASTM C127
Limestone powder	Bulk specific gravity	g·cm^−3^	2.784	ASTM C127
	Moisture content	%	0.23	ASTM C566
	Hydrophilic coefficient	/	0.81	T0353
	Plasticity index	%	3.8	ASTM D4318

**Table 2:** Properties of asphalt binders.

Materials	Properties	Unit	Results	Specifications
Epoxy asphalt	Penetration (25 °C)	0.1 mm	15	ASTM D5
	Softening point	°C	>100	ASTM D36
	Tensile strength	MPa	2.86	ASTM D638
	Elongation at break	%	207	ASTM D638
SBS asphalt	Penetration (25 °C, 5 s, 100 g)	0.1 mm	58	ASTM D5
	Softening point	°C	92	ASTM D36
	Ductility (10 °C, 5 cm/min)	cm	56	ASTM D113
	Density (15 °C)	g/cm^3^	1.029	ASTM D70

**Table 3:** Basic properties of lignin fiber.

Properties	Unit	Results
Fiber length	mm	5.5
pH value	/	7.5
Asphalt absorption rate	%	5.2
Moisture content	%	2

**Table 4:** Volume parameters of asphalt mixtures.

Asphalt Mixture Type	Density(g/cm^3^)	VV (%)	VMA/(%)	VFA (%)	OAC (%)	Stability (kN)	Flow Value (mm)
AC-13	2.414	4.6	15.1	62.9	4.9	13.82	3.6
SMA-10	2.495	3.5	17.1	79.3	6.2	9.33	3.7
EA-10	2.626	1.2	15	92	6.2	64.97	35.7

**Table 5:** Technical indicators of NaCl and Na_2_SO_4_.

Sample	Molecular Weight	Purity (%)	Moisture Content (%)
NaCl	58.44	99.5	0.03
Na_2_SO_4_	142.04	99.3	0.04

## Data Availability

Data will be made available on request.
